# The gut microbiota correlate with the disease characteristics and immune status of patients with untreated diffuse large B-cell lymphoma

**DOI:** 10.3389/fimmu.2023.1105293

**Published:** 2023-02-20

**Authors:** Zhouning Lin, Dan Mao, Changyu Jin, Jiaping Wang, Yanli Lai, Yanli Zhang, Miao Zhou, Qunfang Ge, Ping Zhang, Yongcheng Sun, Kaihong Xu, Yi Wang, Huiling Zhu, Binbin Lai, Hao Wu, Qitian Mu, Guifang Ouyang, Lixia Sheng

**Affiliations:** ^1^ School of Medicine, Ningbo University, Ningbo, Zhejiang, China; ^2^ Department of Hematology, The First Affiliated Hospital of Ningbo University, Ningbo, Zhejiang, China; ^3^ Department of Ultrasound and Medicine, Ningbo Yinzhou People’s Hospital, Ningbo, Zhejiang, China

**Keywords:** diffuse large B-cell lymphoma, gut microbiota, 16s rDNA sequencing, lymphocyte subsets, cytokines

## Abstract

**Background:**

Gut microbiota characteristics in patients with diffuse large B-cell lymphoma (DLBCL) are reportedly different when compared with the healthy population and it remains unclear if the gut microbiota affects host immunity and clinical disease features. This research investigated the gut microbiota in patients with untreated DLBCL and analyzed its correlation with patient clinical characteristics, humoral, and cell immune status.

**Methods:**

Thirty-five patients with untreated DLBCL and 20 healthy controls (HCs) were recruited to this study and microbiota differences in stool samples were analyzed by 16S rDNA sequencing. Absolute ratios of immune cell subset counts in peripheral blood were detected by flow cytometry and peripheral blood cytokine levels were detected by enzyme-linked immunosorbent assay. Relationships between changes in patient microbiomes and clinical characteristics, such as clinical stage, international prognostic index (IPI) risk stratification, cell origin, organ involved and treatment responses were investigated and correlations between differential microbiota and host immune indices were analyzed.

**Results:**

The alpha-diversity index of intestinal microecology in DLBCL patients was not significantly different when compared with HCs (*P*>0.05), nonetheless beta-diversity was significantly decreased (*P*=0.001). *p_Proteobacteria* were dominant in DLBCL, while *p_Bacteroidetes* abundance was significantly decreased when compared with HCs (*P*<0.05). Gut microbiota characteristics were identified that were associated with clinical features, such as tumor load, risk stratification and cell origin, and correlation analyses were performed between differential flora abundance associated with these clinical features and host immune status. The *p_Firmicutes* was positively correlated with absolute lymphocyte values, *g_Prevotella_2* and *s_un_g_Prevotella_2* were negatively correlated with absolute lymphocyte values, T cell counts and CD4 cell counts, while *g_Pyramidobacter*, *s_un_g_Pyramidobacter*, and *f_Peptostreptococcaceae* were negatively correlated with IgA.

**Conclusions:**

Dominant gut microbiota, abundance, diversity, and structure in DLBCL were influenced by the disease, correlated with patient immune status and this suggested that the microecology-immune axis may be involved in regulating lymphoma development. In the future, it may be possible to improve immune function in patients with DLBCL by regulating the gut microbiota, improve treatment response rates and increase patient survival rates.

## 1 Introduction

Diffuse large B-cell lymphoma (DLBCL) is the largest non-Hodgkin’s lymphoma (NHL) subtype, is highly invasive and accounts for about 30% of NHL cases (1). It is a highly heterogeneous tumor in terms of biological features and clinical prognosis, is divided into germinal center B-cell-like (GCB) and activated B-cell-like (ABC) subgroups based on cell origin and depends on different oncogenic pathways with different clinical courses (2). International prognostic (IPI) and age-adjusted IPI (AaIPI) indices can help predict prognosis outcomes (3). As an aggressive NHL, the natural course of DLBCL is relatively short, with immunochemotherapy, based on the CD20 monoclonal antibody such as rituximab substantially improving complete remission (CR) and survival rates in DLBCL patients. However, 30 to 40% of patients will relapse and endure refractory or drug-resistance problems which pose significant challenges for prognoses (4).

With approximately 100 trillion microorganisms in the human gut representing one to three percent of the body’s weight, the gut microbiota is considered the body’s other major organ and affects health *via* an ancient evolutionary symbiotic relationship (5). Many studies have shown that the gut microbiota is linked with diabetes, obesity, cardiovascular disease, inflammatory bowel disease, irritable bowel syndrome and tumors (6–9). The gut microbiota both promotes and inhibits cancer and affects antitumor therapy efficacy. Its mechanism of action toward malignant tumor development may involve inflammation, immune responses, material metabolites and genetic material alteration (10, 11). The microbiota also improves host metabolic capacity and immunoglobulin levels inside and outside the intestinal tract and helps regulate intestinal mucosal immunity (12). Intestinal microecology is not only involved in host digestion, metabolism and energy conversion, but also provides the host with benefits, including strengthening intestinal integrity, regulating intestinal epithelial function, resisting pathogens, supporting lipid metabolism and angiogenesis (13, 14). Intestinal microecology also influences tumor development by interfering with the immune system, inducing genetic mutations, causing chronic inflammation and disrupting the balance between cell proliferation and apoptosis (15).

Although many studies have confirmed that intestinal microecology influences hematological tumor progression and patient prognoses, in DLBCL patients, differences in gut microecological structures between untreated DLBCL onset and healthy populations have been reported (16), while the relationship between intestinal microecology and the immune system and its role in DLBCL development remain poorly understood. More in-depth studies investigating correlations between intestinal microecology, disease characteristics and immune functions in DLBCL patients may provide a basis for disease prevention, early diagnosis, prognostic marker development, and the gut microbiota as a new therapeutic target for DLBCL.

This research used *16S rDNA* sequencing to clarify differences in gut microbiota between DLBCL patients and HCs, by dividing DLBCL into early and advanced-stage groups, high-risk and not-high-risk groups with IPI scores, GCB and non-GCB groups, DLBCL with gastrointestinal involvement (GI) and non-gastrointestinal involvement (NGI) groups, complete remission(CR) and non-complete remission(NCR) groups to compare gut microbiota differences between groups. Correlations between immune indicators and the gut microbiota, which affected patient clinical characteristics, were specifically examined to possibly elucidate microecology-immune axis functions in DLBCL development and develop novel prognostic markers and intervention strategies.

## 2 Materials and methods

### 2.1 Participant characteristics

From September 2018 to November 2021, 35 patients with untreated DLBCL at the Ningbo First Hospital, and 20 matched healthy controls (HCs), were recruited. Patients were required to be free of antibiotics, chemotherapy drugs and other medications that affect the gut microbiota four weeks before stool collection. Exclusion criteria included active gastrointestinal disease, chronic diarrhea or constipation, history of tumor or autoimmune disease. Participants signed an informed consent form. The study was approved by the Ethics Committee of Ningbo First Hospital and registered at the China Clinical Trials Registry (registration number: ChiCTR2100054354).

Based on the World Health Organization (2016) morphological criteria (17), all 35 DLBCL patients were pathologically consistent with DLBCL and patients were immunohistochemically classified into GCB and non-GCB types according to Han’s classification (18). The staging was performed according to disease staging using the Ann Arbor staging system (19) and patient physical status and prognosis information were also assessed using current physical status according to the Eastern Cooperative Oncology Group and IPI scoring systems. Patients were treated with an R-CHOP regimen(rituximab d0, cyclophosphamide d1, doxorubicin d1,vindesine d1, and prednisone d1-5) (20) and chemotherapy dose and frequency schedules were adjusted according to specific conditions. Patients were reviewed by imaging to assess lesions after four cycles of treatment and clinical efficacy was classified as CR and NCR according to international NHL efficacy evaluation standards (21).

### 2.2 Fecal collection and 16S rDNA analysis

Fecal specimens were collected from patients at onset and also from HCs and stored at -80°C in a sterile preservation tube containing an anti-DNA degradation solution.

Following the manufacturer’s instructions, DNA was extracted from fecal samples using the QIAamp Fast DNA Stool Mini Kit (Qiagen, CA, USA). Detection of the isolated DNAs was determined by spectrophotometry (Multiskan™ GO, Thermo Fisher Scientific, USA). The DNA extracts also were checked by 1.5% agarose gel electrophoresis in 1× Tris-Acetate-EDTA buffer. Reaction volumes for polymerase chain reaction (PCR)in a total of 20 μL consisted of 10 μL KAPA HiFi Hot Start Ready Mix (KAPA Biosystems, MA, USA), 2 μL DNA of approximately 30 ng/μL and 1 μL forward and reverse primers of 10 μM. The *16S rDNA* primer sequences were forward 5’-GTGCCAGCMGCCGCGGTAA-3’; reverse 5′-GGACTACNVGGGTWTCTAAT-3′. PCR reaction conditions were configured as required. Products were analyzed using the Qubit 3.0 (Thermo Fisher Scientific, MA, USA) to determine concentrations and then mixed in equal amounts to create a sequencing library. Insert fragments and library molar concentrations were detected and quantified using a QSEP100 (Bioptic, Taiwan) and an ABI7300 fluorescence quantitative PCR instrument (Thermo Fisher Scientific, MA, USA) was used to generate a qualified library for sequencing on the MiniSeq Illumina platform (Illumina, CA, USA). Public access to the original datasets is accessible. This data can be found at https://www.ncbi.nlm.nih.gov/sra/PRJNA906033.

The raw reads were assembled with Flash (22). Primers were removed and clean tags were generated by deleting lower reads with the use of cutadapt (23). The sequences were clustered into operational taxonomic units (OTUs) at 97% similarity using UCHIME in reference mode (24). Representative OTU sequences were aligned in the Silva_132_97_16S database (25) for taxonomic classification using the RDP Classifier (26).

### 2.3 Microbial community composition and differential abundance - statistical analyses

Alpha-diversity estimates included Shannon, Simpson, ACE, and Chao1 by R vegan package. Sample beta-diversity was assessed by weighted and unweighted UniFrac distances and visualized using Principal Coordinate Analysis (PCoA) plots. These analyses were performed in R v3.4.1. Linear discriminant analysis effect size (LEfSe) analysis was performed on the Galaxy platform(www.huttenhower.sph.harvard.edu/galaxy/).

### 2.4 Serum cytokine and immunoglobulin measurements

From participants, 5 mL fasting peripheral venous blood was drawn and interleukin 2 (IL-2), IL-4, IL-6, IL-10, tumor necrosis factor α (TNF-α), and interferon γ (IFN-γ) were measured by enzyme-linked immunosorbent assay (ELISA) (Beckman, CA, USA). Immunoglobulin A (IgA), IgG, and IgM were quantified using an IMMAGE-800 automatic immunochemistry system (Beckman, CA, USA) and accompanying test kits.

### 2.5 Flow cytometry

A two ml peripheral venous blood sample was collected from each participant. Then, 100 μl was mixed with an antibody solution containing20 μl of each of the following antibodies; CD3-fluorescein isothiocyanate (FITC), CD4-allophycocyanin (APC), CD8-phycoerythrin (PE), CD45-peridinin-chlorophyll protein (PerCP), CD16+56-PE, CD19-APC, CD127-PE and CD25- PE-Cy7 according to manufacturer’s instructions. Antibodies were purchased from BD Biosciences (San Jose, CA, USA). Data from 10,000 cells were collected using FACSCanto™ flow cytometry and analyzed using Flowjo 7.6 software.

### 2.6 Statistical methods

Statistical analysis was performed using SPSS23 software. Patient clinical characteristics in groups were compared using the x^2^ test for categorical variables or one-way analysis of variance tests for continuous variables. The t-tests were used for comparisons between groups. Correlations between intestinal microecology and immune indices were analyzed by Spearman’s correlation analysis. Differences were considered statistically significant at P<0.05.

## 3 Results

### 3.1 Baseline characteristics of the study population

Thirty-five patients with DLBCL and 20 HCs were included in this study. In the DLBCL group, there were 21 males and 14 females with a median age of 64 years; nine patients had stage I–II and 26 had stage III–IV disease; 14 cases were GCB and 21 cases were non-GCB type; lesions involved the gastrointestinal tract in nine cases and other extra-nodal organs in 17 cases; lactate dehydrogenase levels were increased in 13 cases and normal in 22 cases; IPI scores were low-intermediate risk in 19 cases and medium-high risk in 16 cases. After four cycles of chemotherapy, 10(29.4%) of 34 evaluable patients achieved CR, 20 (58.8%) achieved partial remission (PR) and 4 (11.7%) had disease progression. The proportion of CR obtained in the mid-term evaluation of this study is slightly lower than the literature data (27), which may be related to the high proportion of patients (74.29%) with high tumor load in stage III-IV among the enrolled patients.

In the HCs group, there were seven males and 13 females with a median age of 57.5 years. There was no statistical difference between these groups in terms of gender and age (**Table 1**).

**Table 1:** Clinical Characteristics of DLBCL patients and healthy controls (HCs).

Clinical Characteristics	DLBCL (n=35)	HCs(n=20)
Number of patients	35	20
Age (years)	34-86(64)	26-78 (57.5)
Patient’s sex (male: female)	21:14	7:13
**Clinical staging**	–	–
Stage I-II	9	–
Stage III-IV	26	–
**Pathological subtype**	–	–
GCB	14	–
non-GCB	21	–
**Involvement site**	–	–
Involvement of the gastrointestinal tract	9	–
Involvement of other extra-nodal organs	17	–
Elevated LDH (≥250u/l)	13	–
**IPI Score**	–	–
IPI <3 points	19	–
IPI ≥3 points	16	–
Ki-67≥80%	21	–
Bcl-2 positive (≥50%)	27	–
Bcl-6 positive (≥50%)	32	–
c-Myc positive (≥30%)	31	–
CD5-positive	9	–
**Efficacy assessment**	–	–
CR	10	–
NCR	24	–

LDH, lactate dehydrogenase; GCB, germinal center B-cell-like; CR, complete remission; NCR, non-complete remission; IPI, international prognostic index.

### 3.2 Altered intestinal microecology in patients with untreated DLBCL

**Figure 1:**
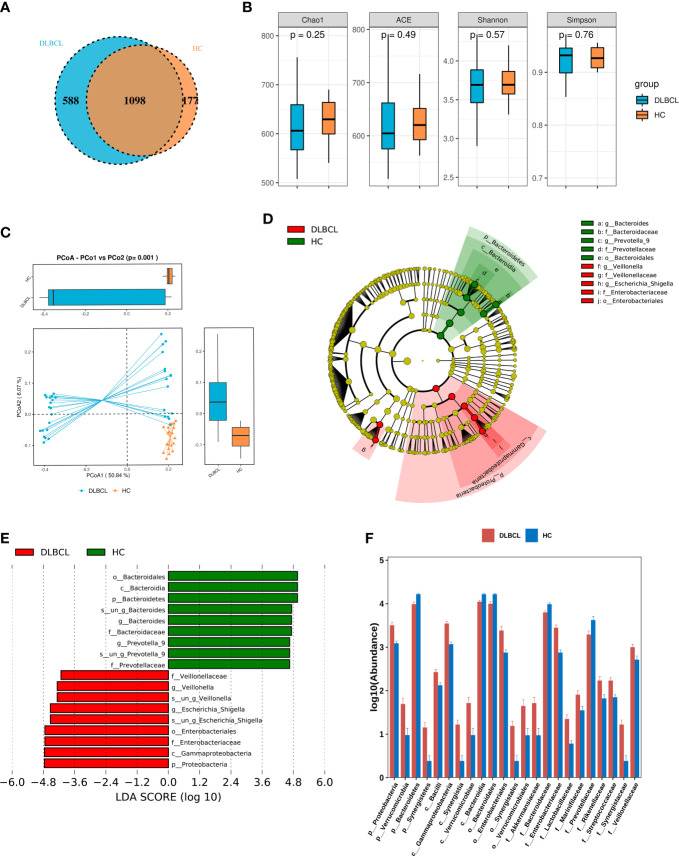
Comparing the gut microbiota between patients with untreated diffuse large B-cell lymphoma (DLBCL) patients and healthy controls (HCs). **(A)** Similarity analysis between DLBCL patients and HCs gut microbiota OTUs. **(B)** Alpha-diversity analysis of DLBCL patients and HCs. **(C)** Beta-diversity analysis of DLBCL patients and HCs. **(D)** Evolutionary relationship diagram: circles arranged radially from inside to outside indicate taxonomic levels from phylum to genus. Yellow nodes indicate taxonomic characters not clearly distinguishable between DLBCL patients and HCs, red nodes indicate richer taxonomic types in the DLBCL group, and green nodes represent richer taxonomic types in the HCs. **(E)** LDA displays species with significant differences in abundance between DLBCL patients and HCs. Red bars indicate taxa enriched in the DLBCL group; green bars indicate taxa enriched in the HCs. **(F)** Relative abundance of DLBCL patients and HCs at the phylum level, order, phylum and family levels.

A total of 55 fecal specimens were analyzed by 16SrDNA gene sequencing. 2,360,952 reads were obtained from all samples, and 25,220 reads were obtained from per sample. OUT analysis showed that the DLBCL group had 588 unique OTUs,the HCs group had 177 unique OTUs, and both groups had 1098 identical OTUs (**Figure 1A**).

The alpha-diversity index of a single-sample can reflect the fecal microbial richness, (ACE and Chao1) and diversity (Shannon and Simpson). Chao1, ACE and Shannon indices were higher in the HCs group when compared with the DLBCL group, but the Simpson index was higher in the DLBCL group, with no statistical differences (*P*>0.05) (**Figure 1B**). Beta-diversity analysis showed that intestinal microbial communities of HCs group were richer than that of the DLBCL group (*P*=0.001) (**Figure 1C**).

The LEfSe analysis found that compared with the HCs group, *p_Proteobacteria*, *c_Gammaproteobacteria*, *o_Enterobacteriales*, *f_Enterobacteriaceae*, and *g_ Escherichia-Shigella* were higher in relative abundance in the DLBCL group, and *p_Bacteroidetes* was lower in relative abundance in the HCs group when the Linear discriminant analysis (LDA) score cutoff was set to 4.0 (**Figures 1D, E**).

At the phylum level, *p_Proteobacteria* (*P*<0.001), *p_Verrucomicrobia* (*P*=0.04), and *p_Synergistetes* (*P*=0.03) abundance was significantly higher in the DLBCL group when compared with the HCs group, while *p_Bacteroidetes* (*P*<0.001) was significantly increased in the HCs group. At the genus level, *g_Escherichia-Shigella* (P<0.001), *g_Veillonella* (*P*< 0.001), *g_Roseburia* (*P*=0.004), *g_Lachnoclostridium* (*P*<0.001), and *g_Alistipes* (*P*=0.002) were significantly more abundant in the DLBCL group when compared with the HCs group, while the opposite was true for *g_Bacteroides* (*P*=0.001), *g_Prevotella_9* (*P*=0.019), and *g_Megamonas* (*P*=0.026) (**Figure 1F**).

### 3.3 The relationship between gut microbiota at DLBCL onset and clinical disease characteristics

**Figure 2:**
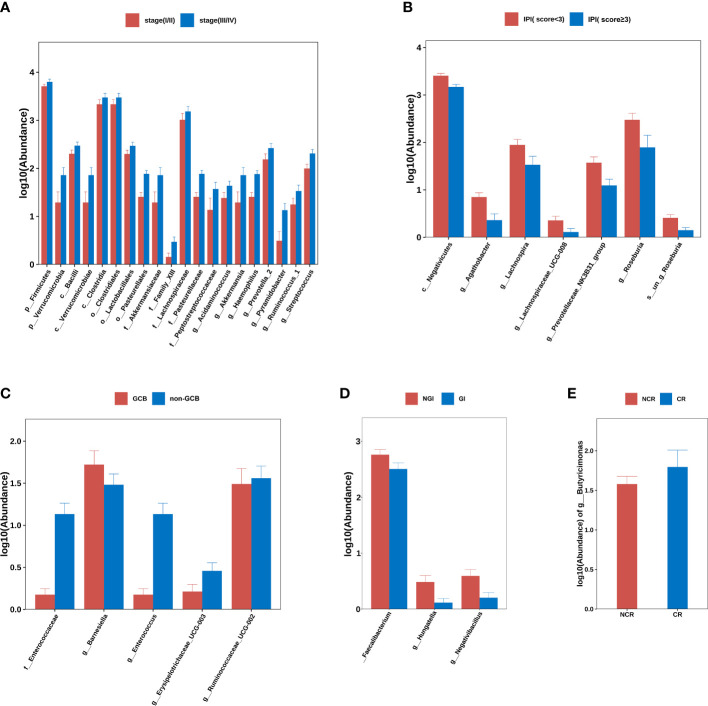
Group differences at phylum, order, family, genus, and species levels. **(A)** stage (I/II) group vs. stage (III/IV) group. **(B)** IPI (score<3) group vs. IPI (score≥3) group. **(C)** GCB group vs. non-GCB group. **(D)** NGI group vs. GI group. **(E)** NCR group vs. CR group. Note: GCB, germinal center B-cell-like. NGI, non-gastrointestinal involvement. GI, gastrointestinal involvement.CR, complete remission. NCR, non-complete remission. IPI, international prognostic index.

Using *Ann Arbor* clinical staging criteria, DLBCL cases were divided into early (I–II) and advanced stage groups (III–IV); no significant differences in alpha- and beta-diversity indices were observed between groups. When differences between two groups were compared at each flora level, it was observed that *p_Firmicutes* (*P*=0.029), *p_Verrucomicrobia* (*P*=0.045), *c_Bacilli* (*P*=0.036), *c_Verrucomicrobiae* (*P*=0.045), *c_Clostridia* (*P*=0.032), *o_Clostridiales* (*P*=0.032), *o_Lactobacillales* (*P*=0.036), *o_Pasteurellales* (*P*=0.001), *o_Verrucomicrobiales* (*P*=0.045), *g_Acidaminococcus* (*P*=0.037), *g_Akkermansia* (*P*=0.045), *g_Haemophilus* (*P*=0.001), *g_Prevotella_2* (*P*=0.048), *g_Pyramidobacter* (*P*=0.019), *g_Ruminococcus_1* (*P*=0.026), and *g_Streptococcus* (*P*=0.008) were all elevated in patients with advanced lymphoma (**Figure 2A**).

The DLBCL cases were divided into IPI < 3 and IPI ≥ 3 subgroups according to IPI scores and there were no significant differences in alpha- and beta-diversity indices between group IPI < 3 and group IPI ≥ 3. When differences in flora between two groups were compared, *c_Negativicutes* (*P*<0.001), *g_Agathobacter* (*P*<0.001), *g_Lachnospira* (*P*=0.013), *g_Lachnospiraceae_UCG-008* (*P*=0.030), *g_Prevotellaceae_NK3B31_group* (*P*=0.009), *g_Roseburia* (*P*=0.005) and *s_un_g_Roseburia* (*P*=0.005) were elevated in abundance in IPI < 3 individuals (**Figure 2B**).

From Han typing, DLBCL cases were divided into GCB and non-GCB types and the differences in alpha- and beta-diversity indices between groups were not significant. By comparing differences in flora levels between groups, *f_Enterococcaceae* abundance was elevated in the non-GCB group (*P*=0.04) (**Figure 2C**).

Based on GI involvement or not, DLBCL cases were divided into GI and NGI groups. No significant differences in alpha- and beta-diversity were identified between groups. By comparing differences between flora levels, we showed that *g_ Faecalibacterium* (*P*=0.04), *g_ Hungatella* (*P*=0.019), and *g_ Negativibacillus* (*P*=0.010) abundance levels were elevated in the GI group (**Figure 2D**).

By comparing the differences in gut microbiota between the CR and NCR groups before chemotherapy, there were no significant differences in alpha- and beta-diversity indices between two groups, but it was found that there were significant differences in *g_Butyricimonas* abundance in the CR group when compared with the NCR group (*P*=0.048) (**Figure 2E**).

### 3.4 Correlation analyses between gut microbiota and immune levels

**Figure 3:**
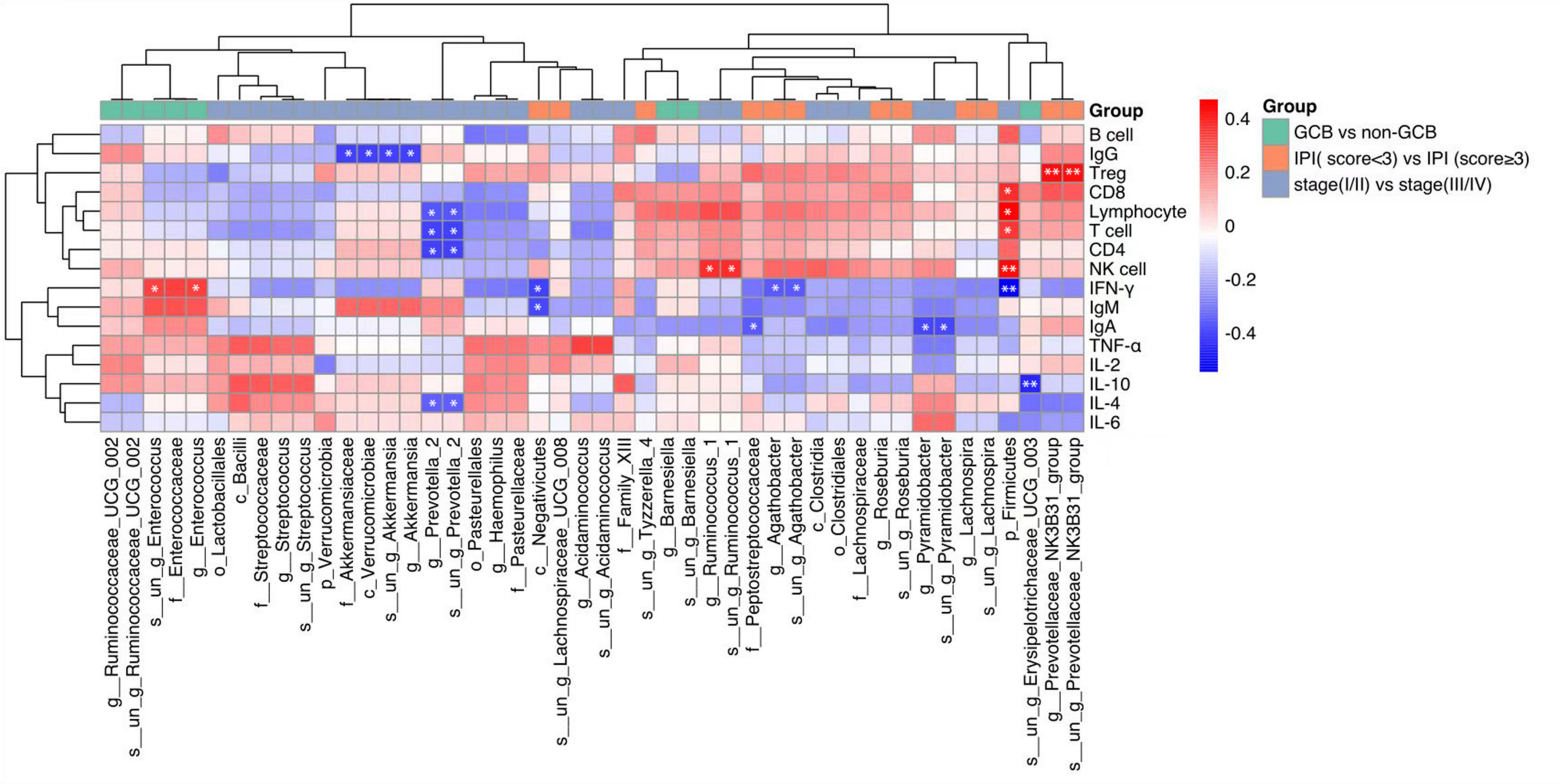
Heat map showing Spearman’s correlations between differential gut microbiota groups and immune indicators. Red indicates a positive correlation and blue indicates a negative correlation. The darker the color, the larger the correlation coefficient. *P<0.05, **P< 0.01. GCB, germinal center B-cell-like; CR, complete remission; NCR, non-complete remission; IPI, international prognostic index.

The correlation between these differential intestinal microflora closely related to the clinical characteristics of DLBCL and the host immune levelwasinvestigated. It was found that *p_Firmicutes* was positively correlated with absolute lymphocyte values, *g:Prevotella_2 and s:un_g_Prevotella_2* were negatively correlated with absolute lymphocyte values, T cell, and CD4 cell counts, while *g_Pyramidobacter*, *s_un_g_Pyramidobacter*, and *f_Peptostreptococcaceae* were negatively correlated with IgA levels (**Figure 3**).

## 4 Discussion

A lot of recent research has revealed a strong relationship between the development of lymphomas and intestinal microecology (28, 29). Using *16S rDNA* gene sequencing of fecal specimens, this study investigated whether intestinal microecology in DLBCL patients was altered. From alpha- and beta-diversity analyses, while gut microbiota alpha-diversity in untreated DLBCL patients was not significantly different when compared with HCs, beta-diversity analysis showed a richer gut microbiota in the HCs group when compared with the DLBCL group. This demonstrated a significant change in intestinal microecology in DLBCL patients.

In the gut microbiota of untreated DLBCL patients, not only was *p_Proteobacteria* abundance significantly higher when compared with HCs, but *c_Gammaproteobacteria*, *o_Enterobacteriales*, *f_Escherobacteriaceae*, and *f_Escherobacteria-Shigella*, which have a continuous evolutionary relationship with *p_Proteobacteria*, were significantly higher when compared with HCs. *f_*Enterobacteriaceae and *g_Escherichia-Shigella* were significantly higher when compared with the HCs group. These microbiotas produce corresponding proteins to affect cell functions, so 75 proteins were specific to *c_Gammaproteobacteria* or *o_Enterobacteriales*, while four including b0354, b1132, b1179, and b3033 were present only in *c_Gammaproteobacteria.* The functions of these specific proteins may help explain the common physiological or biochemical characteristics of these bacteria and future studies may use the molecular characterization of microorganisms to establish gut microbiota markers in DLBCL patients and lead to earlier disease detection.

The *p_Bacteroidetes* abundance was significantly higher in the HCs group when compared with the untreated DLBCL group*. p_Bacteroidetes*, *c_Bacteroidia*, *o_Bacteroidales*, *f_Bacteroidaceae*, *g_Bacteroides*, and *s_un_g_Bacteroides* also demonstrated a continuous evolutionary link at six levels (phylum, class, order, family, genus, and species). It was previously shown that increased *p_Bacteroidetes* levels were closely associated with higher butyrate production (30). Butyrate is a short-chain fatty acid (SCFA) that is produced in the intestine by the bacterial fermentation of dietary fiber (31) and these SCFAs regulate intestinal inflammation by modulating intestinal Treg cell numbers and functions (32, 33). Butyric acid has also been associated with anti-cancer activity in several human tumor cell lines and has acted as a histone deacetylase (HDAC) inhibitor preventing colorectal cancer development (34). A previous study investigated the effects of high-fiber diets on lymphoma and found that butyrate enhanced histone acetylation and pro-apoptotic gene expression, inhibiting lymphoma cell proliferation and apoptosis (35). Additionally, *g_Bacteroides*, as an intestinal commensal bacteria, protects the intestine from pathogens by producing mucin-type O-glycans (36), so it is suggested that increased *p_Bacteroidetes* abundance helps protect the gastrointestinal tract, reduce inflammation and combats lymphoma.

Clinical characteristics such as disease stage, IPI score, and cell origin at disease onset are closely related to disease prognosis (2–4), so the relationships between intestinal microecological alterations at disease onset and clinical features were analyzed to identify associations between gut microbiota, disease onset, disease characteristics and prognosis.

In terms of disease staging, although no significant differences in alpha- and beta-diversity indices were identified between early and advanced groups, among the groups with significantly higher abundance in DLBCL patients when compared with HCs were: *p_Verrucomicrobia*, *c_Bacilli*, *c_Verrucomicrobiae*, *o_Verrucomicrobiales*, *g_Acidaminococcus*, *g_Akkermansia*, *g_Pyramidobacter*, and *g_Streptococcus*; all were elevated in patients with advanced lymphoma, suggesting these floraemay predict a more aggressive clinical course and a worse prognosis for lymphoma.

The organism *Akkermansia muciniphila* is a highly-important and representative human intestinal *p_Verrucomicrobia*, which improves host metabolism and modulates immune responses and is considered a highly promising novel probiotic agent (37). Much evidence now suggests that *A. muciniphila* reductions are associated with several diseases, including obesity, non-alcoholic fatty liver disease, type 2 diabetes, and cardiovascular diseases (38, 39). This bacterium is also believed to enhance antitumor activity and its increased abundance is putatively associated with the reduced incidence of some cancers and the improved efficacy of immune checkpoint inhibitors (40, 41). *A. muciniphila* appears to exert anti-inflammatory effects by suppressing HDACs *via* SCFA production, down-regulating toll-like receptor 4 (TLR4) expression *via* secreted vesicles which regulate the nuclear factor-κB (NF-κB) pathway, and thus inhibiting inflammatory factor release (42). However, *A. muciniphila* itself increases TLR4 expression and elevates cytokines such as IL-6 (42, 43) and was positively associated with IL-6 in a gut microbiota study in elderly debilitated individuals (44). Critically, the IL-6 signaling pathway is a negative predictor in aggressive DLBCL (45), so it is hypothesized that *g_Akkermansia* may increase IL-6 levels and predict a greater tumor load in DLBCL patients, and in this study, the bacterium was elevated in DLBCL patients, especially in those with advanced DLBCL, however it is not possible to infer a causal relationship between the two. The relationship between *Akkermansia* and lymphoma requires more animal studies and clinical trials.

The bacterium *g_Streptococcus* is another that was elevated in this study in patients with advanced lymphoma, and is closely related to colon cancer (46, 47), but its pathogenic mechanisms are unclear. In a study exploring colon cancer and *Streptococcus bovis*, it was found that *S. bovis* promoted proliferation and IL-8 production and promoted precancerous lesion progression in human colorectal adenocarcinoma epithelial cells (48). An *S. bovis* abundance in the intestine of patients with chronic kidney disease may also suggest that renal damage is associated with streptococcal-mediated immune disorders (49). Although *g_Streptococcus* was more abundant in patients with advanced disease when compared with patients with early disease in this study, it remains unclear if the bacterium is involved in DLBCL development and progression, thus further studies are needed.

No significant difference in alpha- and beta-diversity indices was observed between *IPI scores in* not-high-risk and high-risk groups, while comparisons between groups at each taxonomic rank showed that *c_Negativicutes*, *g_Agathobacter*, *g_Lachnospira*, *g_Lachnospiraceae_UCG-008*, *g_Prevotellaceae_ NK3B31_group*, *g_Roseburia*, and *s_un_g_Roseburia* were elevated in IPI< 3individuals. Studies have confirmed that *g_Roseburia* was more closely associated with controlling intestinal inflammation, reducing atherosclerosis, and promoting immune system maturation due to major SCFA production, of which butyrate has a major mediating role (50). *f_Lachnospiraceae* hydrolyzes starch and other sugars to produce butyrate and other SCFAs, thereby promoting immune system maturation (51). Martini et al. (52) extensively analyzed the fecal microbiota in patients with advanced malignancy and found that those with high *Agathobacter* abundance had better progression-free survival, which may be associated with butyrate production. In this study, *g_Roseburia, f_Lachnospiraceae*, and *Agathobacter* flora were elevated in non-high-risk individuals with IPI; these bacteria produce butyrate, therefore it is speculated that they may inhibit lymphoma progression *via* SCFA production.

By comparing differences between GCB and non-GCB groups at all flora levels, *f_Enterococcaceae* abundance was significantly higher in patients with non-GCB. Recently, it was reported that *Enterococcus faecalis* translocated endotoxin to the liver and increased the expression of liver proliferation genes, which are dependent on TLR4-MYD88 signaling, promoting hepato-carcinogenesis (53)The MYD88 activates NF-κB signaling in response to TLR stimulation and also IL-1 and IL-18 receptors and MYD88 mutations are believed to be closely associated with non-GCB type DLBCL development (54). It is therefore hypothesized that *f_Enterococcaceae* may be involved in lymphoma development by acting on MYD88 and activating NF-κB signaling.

Microorganisms are closely associated with the pathogenesis of malignant lymphomas of the gastrointestinal tract. The relationship between *Helicobacter pylori* infection and gastric mucosa-associated lymphoid tissue lymphoma has been established (55), and altered microbiota composition in patients with gastrointestinal follicular lymphoma has also been demonstrated (56). In the present study, there was no significant difference in microbiota composition was observed between the GI and NGI DLBCL patient groups as assessed by diversity indicators. The most well-known species of *g_ Faecalibacterium* is *Faecalibacterium prausnitzii*, which is one of the most important commensal bacteria in the human intestine and one of the main producers of butyrate (57). *F. prausnitzii* and its metabolites can exert an anti-inflammatory effect against colitis in mice, improve intestinal flora dysbiosis, and have the potential to treat inflammatory bowel disease (58, 59). Colorectal cancer patients have fewer butyrate-producing bacteria, including *F. prausnitzii*, and it has been suggested that due to lower butyrate production, the epithelial cell may be more susceptible to being damaged, which may increase the risk of developing cancer (57, 58). In the present study, the abundance of this bacterium was reduced in DLBCL patients with GI, suggesting that the reduction of this bacteria may cause a decrease in intestinal antitumor effects as well as gastrointestinal involvement of lymphoma. However, our study had some limitations due to the small sample size, so further validation in other studies is required.

Several studies have shown the prognostic value of interim [ (18)F]fluorodeoxyglucose positron emission tomography/computed tomography (PET/CT) and can guide subsequent treatment decisions to some extent (27, 60), so patients with PET-CT were evaluated after four cycles of chemotherapy and DLBCL cases divided into CR and NCR group. By comparing the differences in gut microbiota between the two groups before chemotherapy, it was found that higher *g_Butyricimonas* abundance was putatively associated with DLBCL remission after chemotherapy, so it is speculated that the abundance of *g_Butyricimonas* in untreated DLBCL patients could potentially be used as a surrogate predictor of interim outcome and prognosis. Previously, *g_Butyricimonas* occurred in higher abundance in NHL patients at low risk of bloodstream infection (61) and, as SCFA-producing genera, it may influence patient prognoses by modulating the immune system and inflammatory responses (62) and this observation concurred with these findings.

From these analyses, gut microbiota characteristics were identified which were associated with key clinical features such as tumor load, disease risk stratification and cell origin, but it is unclear if these species, with significant effects, affected patient immune functions and disease progression. Several host-microbial interaction studies have shown that relationships between the gut microbiota and the body are mutual and symbiotic. The relationship improves host metabolic capacity, immunoglobulin levels in and outside the gut, and helps regulate intestinal mucosal immunity to some extent. A wide variety of microorganisms in the gut produce antitumor substances that remove malignant proliferating cells before they form tumors (63). The gut microbiota can also influence the activity of the cytotoxic T lymphocyte-associated antigen-4 or programmed death (PD)-1/PD ligand (PD-L) 1 axis by affecting anti-cancer immuno-surveillance and thus immuno-checkpoint inhibitor therapy efficacy (64). Sivan et al. reported that the combination of oral *Bifidobacterium* with PD-L1 virtually eliminated tumor growth. Previous studies have shown that microbial immune interactions may affect tumor occurrence, development, and prognosis, but in patients with lymphoma, it is unclear if disease related intestinal microecological variations are closely related to host immunity, so correlations between differential flora and host immune indices were investigated. Patients with advanced-stage DLBCL tended to have a higher abundance of *g_Prevotella_2* and *s_un_g_Prevotella_2*. This abundance is often related to lower absolute counts of total lymphocytes, CD3+T lymphocytes, and CD4+T cells, so it was hypothesized that *g_Prevotella_2* and *s_un_g_Prevotella_2* may contribute to lymphoma development by reducing CD4+ Th cell and lymphocyte numbers, weakening the host’s anti-lymphoma immunity. *Firmicutes* abundance was positively correlated with absolute lymphocyte counts and it was previously reported that absolute lymphocyte counts in lymphoma patients were closely related to patient prognosis (65). However, this study showed that high *Firmicutes* abundance correlated with disease progression, which suggested that *Firmicutes*, as a pro-inflammatory gut microbiota, did not produce effective anti-lymphoma immune responses although it increased lymphocyte numbers, so *Firmicutes* may be involved in immune depletion associated with chronic inflammation.

The most abundant type of antibody, IgA, serves as the first line of defense for the immune system on mucosal surfaces. Recent studies suggested that changes in microbiota diversity could modulate the IgA-microbiota axis and affect antitumor immunity by altering cancer development risks and modulating responses to immunotherapy. IgA exerts antitumor or pro-tumor effects on different tumor types and may be influenced by tumor type, environmental, and host factors (66). However, the role of IgA in lymphoma is unclear. In this study, *f_Peptostreptococcaceae*, *g_Pyramidobacter* and *s_un_g_Pyramidobacter* showed negative correlations with IgA, while high *g_Pyramidobacter* abundance was associated with a more advanced disease stage, suggesting this bacterium may affect anti-tumor immunity *via* IgA. Studies should be conducted to disseminate its role in anti-lymphoma immunity.

## 5 Conclusions

This study described gut microbiota characteristics in *de novo* DLBCL patients for the first time and identified flora markers closely related to patient clinical characteristics. These markers included disease stage, cell source, IPI risk stratification, and immunochemotherapy responses. It also showed correlations between structural flora variations and host immunity, and provided new insights into the intestinal microecology immune axis during DLBCL development. However, further work is required to uncover relationships between intestinal microecology and DLBCL development. In future studies, the specific mechanisms of differential and dominant flora development in DLBCL must be characterized to determine new disease biomarkers and develop new therapeutic strategies.

## Data availability statement

The original contributions presented in the study are publicly available. This data can be found here: https://www.ncbi.nlm.nih.gov/bioproject/PRJNA906033.

## Ethics statement

Written informed consent was obtained from the individual(s) for the publication of any potentially identifiable images or data included in this article.

## Author contributions

LS and YZ obtained funding. ZL and DM drafted the manuscript. ZL and CJ made the figures and tables. DM, JW, YZ and PZ analysed data. CJ, MZ, QG and YS collected and processed specimens. KX, YW, BL, HW collected clinical data. GO, HZ and LS designed the study. LS and GO revised the manuscript. All authors contributed to the article and approved the submitted version.

## Supplementary material

Supplementary Figure 1
**(A)** Alpha-diversity analysis of stage (I/II) and stage (III/IV). **(B)** Beta-diversity analysis of stage (I/II) and stage (III/IV).

Supplementary Figure 2
**(A)** Alpha-diversity analysis of IPI (score<3) and IPI (score≥3). **(B)** Beta-diversity analysis of IPI (score<3) and IPI (score≥3).

Supplementary Figure 3
**(A)** Alpha-diversity analysis of GCB and non-GCB. **(B)** Beta-diversity analysis of GCB and non-GCB.

Supplementary Figure 4
**(A)** Alpha-diversity analysis of NGI and GI. **(B)** Beta-diversity analysis of NGI and GI.

Supplementary Figure 5
**(A)** Alpha-diversity analysis of NCR and CR. **(B)** Beta-diversity analysis of NCR and CR.
